# Opsoclonus myoclonus syndrome in a patient with Japanese encephalitis: a case report

**DOI:** 10.1186/s13256-017-1454-5

**Published:** 2017-10-23

**Authors:** Subatharshini Sountharalingam, H. M. M. T. B. Herath, Dharshana Wijegunasinghe, Sunethra Senanayke

**Affiliations:** 1 Medical Department, National Hospital of Sri Lanka, Colombo, Sri Lanka; 2 Neurology Department, National Hospital of Sri Lanka, Colombo, Sri Lanka

**Keywords:** Japanese encephalitis, Opsoclonus myoclonus syndrome

## Abstract

**Background:**

Opsoclonus myoclonus syndrome is a rare neurological disorder that usually manifests as a paraneoplastic phenomenon. Although some viruses are reported to cause this condition, opsoclonus myoclonus syndrome by Japanese encephalitis has not been reported previously.

**Case presentation:**

Here we present the case of a 31-year-old Sri Lankan woman who presented with fever, altered level of consciousness, opsoclonus, and facial myoclonus. She was diagnosed as having Japanese encephalitis based on cerebrospinal fluid and serum Japanese encephalitis-specific immunoglobulin M antibody and characteristic magnetic resonance imaging abnormalities. She was given intravenously administered methylprednisolone pulses (1000 mg per day) for 5 days. With this she improved gradually with reduction in opsoclonus and myoclonic movements. Her limb muscle power and speech also improved slowly.

**Conclusions:**

We intended to highlight the fact that opsoclonus myoclonus syndrome can be a feature of infection with Japanese encephalitis and that it can be added to the list of viruses which cause opsoclonus. Currently there is no well-accepted treatment for opsoclonus myoclonus syndrome and intravenously administered methylprednisolone pulses and immunosuppressants can be used successfully in these patients for early recovery.

## Background

Japanese encephalitis (JE) is a mosquito-borne viral encephalitis caused by a *Flavivirus* closely related to West Nile and St. Louis encephalitis viruses [1]. Humans are an incidental dead-end host while pigs and birds are the amplifying hosts. The culicine mosquito species *Culex tritaeniorhynchus* and *Culex gelidus* are the principal vectors. However, some other culicine mosquito species such as *Culex vishnui*, *Culex pseudovishnui*, and *Culex fuscocephala* are also responsible for the transfer of the virus to humans from amplifying hosts [1, 2]. A majority of infections are asymptomatic and only less than 1% manifest as encephalitis [1]. Nearly 35,000 to 50,000 cases of JE are reported to the World Health Organization (WHO) each year, resulting in an estimated 10,000 to 15,000 deaths annually. The case fatality rate varies between 5 and 30%, while 30 to 50% of survivors have significant neurological sequelae [1].

The clinical course is divided into prodromal phase, acute encephalitic stage, and late phase. The prodromal phase starts before the onset of encephalitis, with fever and constitutional symptoms. Altered sensorium, nuchal rigidity, and abnormal movements characterize the encephalitic stage. Recovery or persistence of central nervous system signs occurs in the late phase [1]. Cases of opsoclonus myoclonus syndrome (OMS) caused by JE have not been reported previously. Here we discuss a patient with JE who presented with OMS.

## Case presentation

A 31-year-old previously healthy Sri Lankan woman from southern parts of Sri Lanka presented with fever for 3 days and altered level of consciousness for 1 day. The fever was associated with headache and myalgia and she did not have nausea, vomiting, or skin rashes. On admission to our hospital she was afebrile and nuchal rigidity was present. She was conscious but aphasic. Her Glasgow Coma Scale was 11/15 (E 4, V 1, M 6). There were involuntary conjugate fast eye movements in all the directions of gaze without a saccadic interval, suggestive of opsoclonus. There were no ophthalmoplegia or other cranial nerve palsies. Her pupils were equally reacting to light. Fundoscopy revealed papilledema. There were involuntary twitching movements on the right side of her face, mandible, and tongue, which increased with movement and disappeared during sleep. Both upper and lower limb tone were normal and she was able to move her limbs against gravity, but not against resistance (power 3/5). The deep tendon reflexes were present. Bilateral plantar responses were flexor. Her other vital signs were stable with a heart rate of 68 beats per minute (bpm), blood pressure of 130/80 mmHg, and blood oxygen saturation (spO_2_) on air was 96%. All other system examinations were unremarkable.

**Fig. 1:**
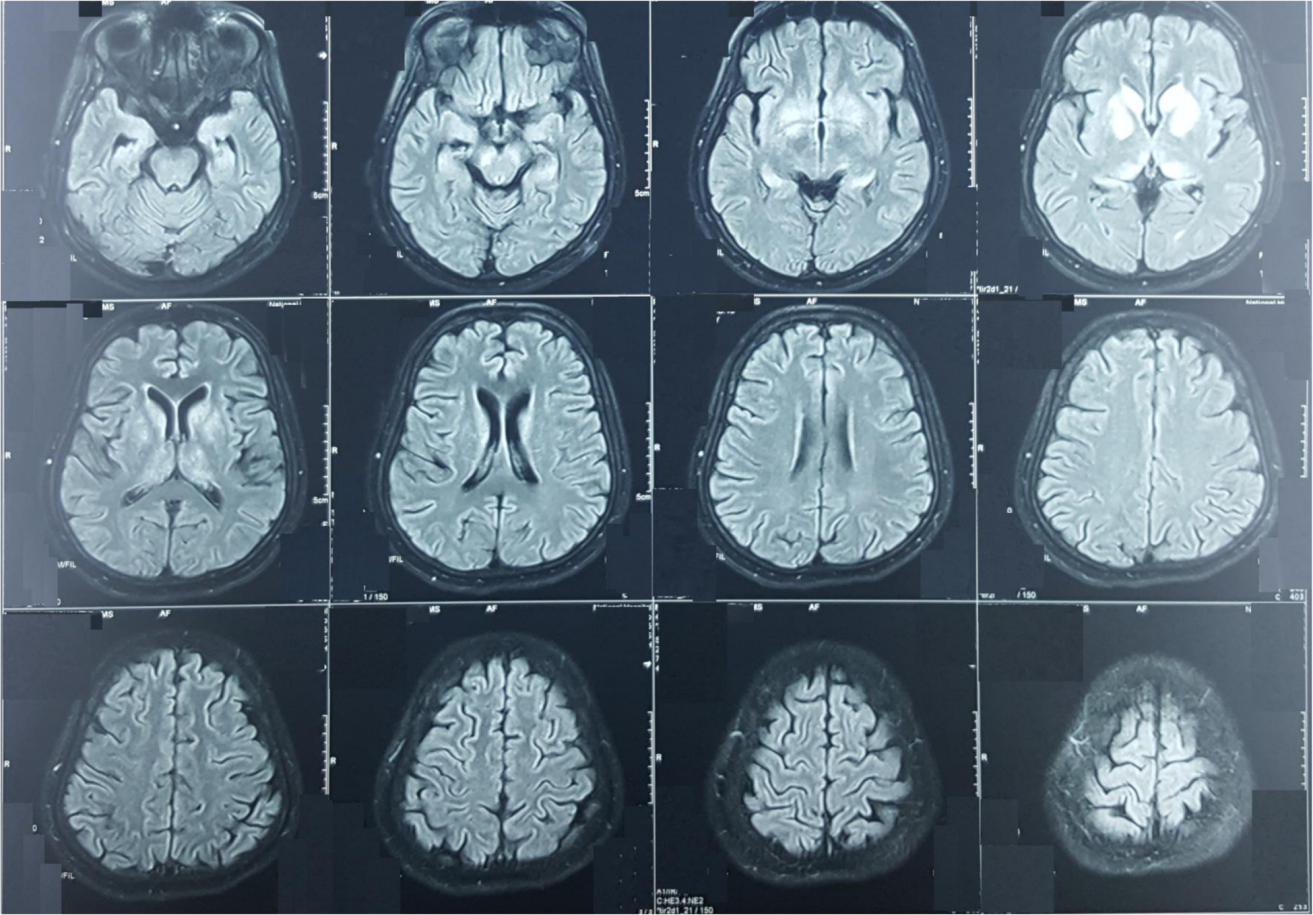
Magnetic resonance imaging of the patient’s brain showing symmetrical bilateral high signal intensities in basal ganglia, caudate head, and midbrain in fluid-attenuated inversion recovery

**Fig. 2:**
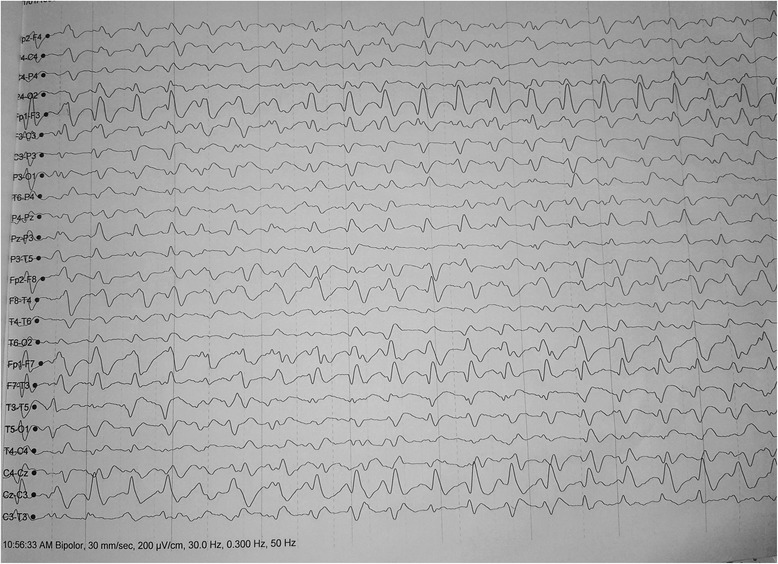
Electroencephalogram showing bilateral periodic lateralized epileptiform discharges

Her full blood count revealed a neutrophil leukocytosis: white blood cells, 14 × 10^3^/μL; neutrophills (N), 78%; lymphocytes (L), 11%; eosinophills (E), 02%; basophills (B), 5%; platelets, 280 × 10^3^/μL; and hemoglobin, 13 g/dl. Her erythrocyte sedimentation rate was 66 mm in the first hour but her C-reactive protein was less than 6 mg/dl. Her serum electrolytes and renal and liver profiles were normal: sodium (Na), 133 mmol/l; potassium (K), 4.5 mmol/l; serum calcium, 2.3 mmol/l; magnesium, 0.99 mmol/l; aspartate aminotransferase (AST), 40 U/l; and alanine aminotransferase (ALT), 47 U/l. Her thyroid stimulating hormone was 0.34 IU/L. Blood and urine culture, blood film for malaria parasite, rheumatoid factor, antinuclear antibody, and human immunodeficiency virus serology were negative. Thyroid microsomal antibody was less than 10 IU/ml and *N*-methyl-D-aspartate (NMDA) receptor antibody was also negative. A non-contrast computed tomography of her brain showed cerebral edema. Cerebrospinal fluid (CSF) opening pressure was 180 mmH_2_O and full report showed high level of proteins of 130 mg/dl with 60 lymphocytes/mm^3^. Polymorphs and red blood cells were absent in CSF. CSF glucose was 3.4 mmol/l (corresponding random blood sugar was 5.5 mmol/l). Herpes simplex virus polymerase chain reaction in CSF was negative. IgM for JE became positive in both serum and CSF. Magnetic resonance imaging (MRI) of her brain showed symmetrical bilateral high signal intensities in basal ganglia, head of the caudate, and midbrain in T2 and fluid-attenuated inversion recovery (FLAIR) without diffusion restriction (Fig. 1). Serial electroencephalograms (EEGs) were done which showed various epileptiform discharges. Initial EEG showed bilateral periodic lateralized epileptiform discharges (Fig. 2) and the second EEG after 2 days showed left-sided lateralization with background slowing.

She was given supportive care and once JE was confirmed we administered methylprednisolone pulses intravenously (1000 mg per day) for 5 days. With this, she improved gradually with reduction in opsoclonus and myoclonic movements. Her limb muscle power and speech also improved slowly. After approximately 2 weeks of the disease duration, epileptic discharges and background slowing in an EEG showed improvement. During the course of her illness she became rigid and a quarter of a tablet of levodopa-carbidopa (250/25 mg) was started at a frequency of three times a day to alleviate the extrapyramidal symptoms.

## Discussion

Viral encephalitis classically causes fever, headache, altered sensorium, neuropsychiatric manifestations, catatonia, and abnormal movements such as orolingual tremors, myokymia, and lip smacking, and mandibulo-faciolingual tremors are also reported [3, 4]. Even though OMS is not commonly caused by infections, there are case reports of viral encephalitis which had caused OMS such as varicella zoster, West Nile encephalitis [5], and mumps [6]. OMS after influenza vaccination was also reported [7] and in India OMS due to malaria had been reported [8].

OMS also known as myoclonic encephalopathy (Kinsbourne syndrome) and “dancing eyes-dancing feet syndrome”, is a rare neurological disorder that occurs at a prevalence of 1 in 10,000,000. It usually occurs in children with neuroblastoma as a paraneoplastic manifestation and in adults due to underlying lung cancer and gynecological malignancies. Toxins and autoimmune diseases like Hashimoto encephalopathy are known to cause OMS as well [9]. Opsoclonus is defined as chaotic, conjugate, multivector, back-to-back, saccadic eye movements without intersaccadic latency. It is also called saccadomania [9]. It is thought to occur due to damage of the omnipause cells in the pontine raphe nucleus [9]. Our patient had opsoclonus and involuntary twitching movements of the right side of her face suggestive of myoclonus. Ataxia and cerebellar involvement could not be assessed in our patient.

Diagnosis of JE depends on imaging and CSF analysis. Characteristic MRI appearance is T2 and FLAIR hyperintensities in thalamus, basal ganglia, caudate, and midbrain bilaterally. Although CSF pressure is elevated in approximately 50% of cases, it was normal in our patient. A CSF full report showed elevated protein with CSF lymphocytosis typical of JE. JE-specific IgM antibody was positive in CSF as well as in serum in the second week of illness. Ultrasound imaging of her abdomen and a chest X-ray that were done to exclude underlying malignancies were normal. Hashimoto encephalopathy and autoimmune encephalitis also can cause a similar clinical picture but thyroid peroxidase antibody and NMDA receptor antibody were negative in our patient.

Various therapies have been tried successfully in patients with OMS and they include corticosteroids, intravenously administered immunoglobulin, immunosuppressants, plasma exchange, rituximab, adrenocorticotropic hormone (ACTH), and clonazepam. In our case, a significant improvement was observed with intravenously administered methylprednisolone pulses over a short period of time. Parkinsonism features improved dramatically after a small dose of levodopa-carbidopa.

## Conclusions

We intended to highlight that OMS can also be a feature of JE and that this virus can be added to the list of viruses that can cause OMS. Currently there is no well-accepted treatment in OMS and intravenously administered methylprednisolone pulses and immunosuppressants can be used successfully in these patients for early recovery.

## Abbreviations

ACTH: Adrenocorticotropic hormone
ALT: Alanine aminotransferase
AST: Aspartate aminotransferase
bpm: Beats per minute
CSF: Cerebrospinal fluid
EEG: Electroencephalogram
FLAIR: Fluid-attenuated inversion recovery
JE: Japanese encephalitis
K: Potassium
MRI: Magnetic resonance imaging
Na: Sodium
NMDA: *N*-methyl-D-aspartate
OMS: Opsoclonus myoclonus syndrome
SpO_2_: Blood oxygen saturation
WHO: World Health Organization
